# The new chiropractic

**DOI:** 10.1186/s12998-016-0108-9

**Published:** 2016-06-30

**Authors:** Bruce F. Walker

**Affiliations:** Discipline of Chiropractic, School of Health Professions, Murdoch University, Murdoch, Western Australia 6150 Australia

**Keywords:** Chiropractic, Legitimacy, Evidence based practice, The new chiropractic

## Abstract

**Background:**

Physical manipulation and manual therapies are thousands of years old. The most popular western world iteration of these therapies is delivered by chiropractors. It can be argued that the collective public health benefit from chiropractic for spinal pain has been very substantial, however as chiropractic has transitioned from craft to profession it has encountered many internally and externally driven machinations that have retarded its progress to a fully accepted allied health profession. This article sets out a ten point plan for a new chiropractic that will achieve full acceptance for this troubled profession.

**Discussion:**

This article is based on a keynote speech known as the *FG Roberts Memorial Address* delivered on October 10, 2015, in Melbourne, Australia at the Chiropractic & Osteopathic College of Australasia and Chiropractic Australia national conference.

The ten point plan consists of the following: improving the pre-professional education of chiropractors, establishing a progressive identity, developing a special interest for the profession, marginalising the nonsensical elements of the profession, being pro-public health, supporting the legitimate organised elements of the profession, improving clinical practice, embracing evidence based practice, supporting research and showing personal leadership.

**Conclusion:**

Adherence to this fresh ten point plan will, over time, see the chiropractic profession gain full legitimacy in the allied health field and acceptance by other health providers, policy makers and the public at large.

## Background

Manual therapies including manipulation have been used for centuries, indeed even thousands of years [1]. Many cultures have practitioners that administer these manual therapies for musculoskeletal pain and they can be variously classed as traditional healers including bone setters in England, Kung Fu masters in Asia, and more lately osteopaths, chiropractors and physiotherapists in the western world [1, 2]. Traditionally these manual therapies were taught father to son, mother to daughter, or master to apprentice and in some cultures this still occurs [1–3]. Although this article concentrates on chiropractic, much of what is discussed has implications for other manual therapy professions.

### The “good” and the “bad”

Chiropractic is 120 years old and in that time has progressed from a full alternative medicine concept to be part of complementary medicine and in some jurisdictions it has primary care status. Some argue that historically chiropractic has elements that are consistent with religion [4]. Regardless, what can be said is that the profession’s history, conduct and overall contribution is chequered by “good” and by “bad”. The “good” can be summed by recognising over a century of improvement to public health by improving pain and disability in countries where chiropractic is practised. It can be asserted that this has provided significant economic savings and improved productivity. There has been important global benefit from chiropractic treatment and although this has not been estimated it must be very substantial. The profession can hold its head up high when reflecting on this positive population benefit over time.

Before moving on to the “bad” aspects practiced by a minority within chiropractic I contend that the global “good” produced by the profession far outweighs the “bad” and this point should not be ignored when reading this article. The “bad” side of the profession has been the subject of much controversy, publicity and indeed previous F.G. Roberts addresses [5, 6]. Table 1 lists some of these “bad” practices that continue to cause significant reputational damage to the profession.

**Table 1:** Aberrant practices that cause significant reputational damage to the profession

Adherence to a flawed chiropractic ideology centring on innate intelligence and vitalism	Claims of cures for visceral and other non-musculo-skeletal conditions
Anti-vaccination propaganda	Anti-drug and anti-medicine propaganda
Anti-physiotherapy sentiments	Misleading and deceptive advertising
Open plan clinics where multiple people are treated in the same room fully dressed	Unscrupulous contracts of care
Over-servicing	Obligatory full spine x-rays
Use of the term “subluxation” as a valid diagnosis	Unnecessary treatment of babies
Biologically implausible diagnostic tests and therapies	Unfounded claims of decreased immunity from “subluxation” and increased immunity from chiropractic treatment
Life time chiropractic care in the name of “wellness”	An unhealthy disregard of clinical research, evidence based practice, and non-specific treatment effects including natural history and the placebo effect.

### Where is the profession at this moment in time?

Currently there are many countries that have registration and licensing for chiropractors and educational programs with somewhat differing standards and emphases, housed mostly in private colleges but with a minority in government funded university programs. There is some limited private health funding for patient consultations, minimal public funding for patients, very little hospital access, and research that can only be described as embryonic. The profession by and large graduates competent manual therapists who contribute well to their communities and are good professional citizens, but there are still aberrant elements with a profound retrograde ideology. These individual and organised elements have caused the profession untold reputational damage and continue to do so. As a consequence the professions reputation is commonly poor among other health professions with whom it is compared and its approval is variable within the community at large.

So with all this in mind how does the profession progress to make it equal and worthy partners in the health arena? A profession where it can command a healthy respect by others in the health sector, policy makers and patients alike? A profession where it is welcomed as a legitimate partner in health care delivery?

To achieve this the profession has a stark choice. It can maintain the status quo, keep on the current track and forever be regarded as alternative or complementary practitioners who are just “different”. Or it can develop a vision for a new chiropractor who has the recognised attributes of a fully worthy and functioning allied health professional and contributes more in their respective health field than others do. This article aims at achieving this goal.

To advance the profession globally over the next generation and to achieve the vision set out above a ten point plan is proposed. It is recognised that even if the plan is adopted it will take this significant additional time of a generation to succeed. Hopefully young members of the profession are sufficiently motivated to take on this cause and see it through. If they do, the reputation of the profession will be enhanced to the extent that chiropractors and chiropractic will be seen as legitimate partners in the health care delivery system. It is reasonable to speculate that this goal matches the aspirations of our younger professionals as they look forward to a long and virtuous career.

## Discussion

A ten point plan to fully legitimise the chiropractic profession.

1. There is a need to improve pre professional education for chiropractors.
  *Universities or private colleges?*
  Chiropractic education should where possible be conducted at universities and this does not mean small single purpose institutions that are deemed universities in name only. Why is this recommended? Primarily because unlike some private colleges, government funded universities insist on intellectual evidence based rigour in their learning and teaching and importantly require staff to be research active. Chiropractic courses need to have an underpinning pedagogy that insists that content is taught in the context of the evidence and that students obtain the necessary training to question and critically appraise.
  Private colleges and programs such as we see in the USA and other countries do not usually require all staff to produce high quality research nor can they typically grant higher degrees by way of research such as Doctorates in Philosophy (PhDs). It can be argued that this phenomenon is partially the reason for the poor research output legacy from the USA that has left the profession in a parlous state regarding the body of knowledge that underpins what it does and does not do. It is worth reflecting on the increase in knowledge the profession would now enjoy if all chiropractic academic staff worldwide were research active.
  *Accreditation problems*
  Underpinning chiropractic education is program accreditation and this is also in need of review particularly where vitalistic subluxation based courses have been legitimised by the accreditation process. The question needing to be posed is *“if colleges espouse to graduate subluxation based chiropractors how can they legitimately achieve accreditation?”* I postulate that it is self-evident that there must be something wrong with an accreditation system that allows this to happen regardless of how worthy the institution is in other ways.
  *Hospital training*
  Chiropractic education should also involve specifically relevant hospital access or work experience such as hospital rounds so that students can observe patients that are truly unwell and observe the signs and symptoms taught in their theory classes. Hospital rounds would also allow chiropractic students to interact with other health providers and increase the likelihood of legitimate partnership and respect between health professions.
  *Who should teach chiropractic students?*
  The faculty of teachers of chiropractic students’ needs to be multi-disciplinary with medical doctors, physiotherapists and other allied health personnel involved in their education. Simply put teachers of chiropractic students should be the most skilled and experienced in the content area involved.
2. There is a need to establish a progressive identity.
  Chiropractors need to become solely musculoskeletal practitioners with a special emphasis on spinal pain. If the profession becomes the world’s experts in this area it will command the respect deserved. Importantly it will not be seen as a collective of alternative medicine practitioners with a strange belief system and a large minority displaying derision of science and medicine. The “spine care model” [7] is a means of developing chiropractic cultural authority and relevancy. The benefits that will accrue from this decision alone are potentially immense including helping to integrate chiropractic care into the mainstream health system while still retaining self-identity for the profession. It is important that our identity as spinal pain experts be driven by current and future evidence. It is this evidence that will help shape our future identity and not ideology or dogma.
3. The profession should develop a generalised special interest.
  In the 1980s a research arm of the psychology profession in the USA developed a special interest in health statistics [8]. This interest made them a pre-eminent group in a field which stood them apart from other health professions. Their work contributed to the health statistics field generally and assisted psychology to become worthy allied health partners. Chiropractic as a profession should also develop a special interest area in the health sciences that can make a worldwide contribution to other related health sciences. This could be either research based or clinically based or indeed both. Some possibilities are: the further development and refinement of evidence based practice, improved posture through motor control, musculoskeletal care for the aged and elderly, improving bone density or the very important area of translating research into practice via implementation science. Whatever chosen we need to develop a special interest that sets us apart as experts in a distinctive area.
4. Marginalisation of the nonsensical elements within the profession.
  As professionals chiropractors should not tolerate colleagues or leadership in the profession who demonstrate aberrant ideas. If colleagues transgress the boundaries or professionalism they should be reported to authorities and this should be followed up with action by those authorities. It is important to reject the notion of embracing the margins of diversity within the profession. If an idea is nonsensical practitioners should express their opinion and make a stand, otherwise their silence will be regarded as consent. Insist that professional chiropractic organisations take a public stand against biologically implausible notions and theories, illogical ideas, dangerous practices and be prepared to report even likable but misguided individuals. It is not treason to seriously question abnormal and implausible ideas within the chiropractic profession. In the short term the consequences may be cathartic but they will ultimately lead to positive change.
5. The profession and individual practitioners should be pro public health.
  It is important to speak up openly in favour of evidence-based public health measures and to join public health associations and agencies. Chiropractic can and should get involved in health promotion and disease prevention especially in two main areas. First, where there is a synergy with musculoskeletal disorders such as osteoporosis, falls prevention, the ageing spine, ergonomic design of furniture and work stations to name a few; and second to actively counter the disinformation perpetrated by rogue elements within the profession. For example, chiropractors promoting anti-vaccination views need to be countered and also those in the profession who seek to medicalise infancy by “diagnosing” infants with notional spinal manipulable lesions. In this way we will nullify the destructive reputational damage caused by anti-public health advocates in our profession and in turn bolster public health and indeed chiropractic’s reputation through advocacy in these areas.
6. Support legitimate organised elements of the profession.
  Practitioners should support and become involved in chiropractic organisations that are clearly ethical and evidence based and add value to them. It is important not to expect others to do this for you. Be wary of organisations that just pay lip service to ethical evidence based practice (EBP). Ultimately these groups will be judged by their deeds and not their words. As a way forward we should seek contrition and apologies from organisations that have damaged the reputation of the profession over decades. Indeed, unambiguous statements from chiropractic organisations supporting science and evidence in practice are overdue.
  While it is important to support progressive professional organisations it is also worth demanding service and accountability from them. As an example chiropractic organisations should be encouraged to market and advertise the profession at large in a non-controversial manner with professional advertisements and media that reflects the truth and has at its heart the public good. Regular collective professional advertising of the benefits of chiropractic for back pain, for example, is a worthy undertaking but the advertisements or media offerings must be evidence based.
7. The profession should strive to improve clinical practice.
  Chiropractors contribute to the public health by the aggregated benefit of positive outcomes to health from their clinical practices. In this domain the profession should examine all aspects of what it does and why it is done. Currently heterogeneity in chiropractic practice is common but it will not always be that way. In the future more and more evidence will inform our history taking, examination procedures, selection of diagnostic tests, the therapies chosen and the advice given. It is very likely that the future chiropractor will be more efficient, safer and more effective in clinical practice for those with spinal pain than those of today. The profession should not be afraid of the change demanded by new evidence. Indeed, chiropractors should be prepared to alter how they as individual practitioners communicate with patients, the diagnostic tests used or ordered, the prevention strategies recommended and the therapies administered including the use of medication and other interventions that are synergistic with musculoskeletal disorders. With respect to medication there has been a significant shift in attitudes within the profession regarding the use of medication in chiropractic practice [9, 10]. Notwithstanding restrictive laws in various jurisdictions regarding medication prescribing rights what will matter in the future is cost-effectiveness not an anti-drug dogma. Where restrictive practice laws relating to chiropractors prescribing medication exist the profession should seek to overturn them.
  A concluding point on clinical practice is that chiropractors should, where possible, seek to work in multi-disciplinary environments as this will mitigate the intellectual isolation suffered by a proportion of the profession.
8. The profession should embrace evidence based practice.
  EBP is the amalgam of best scientific evidence plus clinical expertise plus patient values and circumstances. So what could be missing from this equation? It is clear that in the opinion of a sizable minority of the profession the elements that are missing are “practitioner ideology” and “practitioner values and circumstances”. These additional self- serving and dangerous notions should not be entertained. The adoption of evidence based practice is critical to the future of chiropractic and yet there is resistance by elements within the profession. Soft resistance occurs with attempts to change the name of “Evidence-based practice” (EBP) to “Evidence-informed practice” (EIP). It is worth noting that currently there are over 13,000 articles listed in PUBMED on EBP but less than 100 listed on EIP. So why are some of our profession so keen to use this alternate and weaker term?
  Hard resistance against EBP occurs where it is stated that the best evidence is that based on practice experience and not research. This apparently is known as Practice Based Evidence (PBE) and has a band of followers. It is worth illustrating the difference between EBP and PBE with an example from the literature. Table 2 is taken from the study by Olafsdottir et al. in 2001 (with permission). The study is a randomised trial looking at chiropractic treatment for infantile colic [11]. The current evidence about infantile colic and chiropractic is conflicting but this study is useful for demonstration purposes.

**Table 2:** Hours of crying before and during the treatment period of eight days for those infants whose parents managed to complete the diary appropriately

	Treatment group	Controls	*p* value
Pre-treatment	5.1 (3.0) (n = 41)	5.4 (3.2) (n = 31)	0.612
At first visit	4.2 (2.7) (n = 42)	3.9 (2.5) (n = 33)	0.612
At second visit (day 3–6)	3.4 (2.7) (n = 42)	3.2 (2.5) (n = 33)	0.602
At third visit (day 8)	3.1 (2.7) (n = 42)	3.1 (2.7) (n = 33)	0.982

Results expressed as mean (SD); *p* values calculated by Student’s *t* test

Reproduced with permission
  The left (treatment group) column of the table shows a decrease in hours of baby crying after chiropractic treatment and could be said to represent “practice based evidence”. It is the experience practitioners often have when patients (or in this case parents) report improvement in the clinical setting. However, a review of the table’s right (controls) column shows a similar decrease in hours of crying for the babies who did not receive any treatment. Importantly in this trial the parents were not aware of which treatment arm the babies were randomised to. So when the right and left columns are combined we have the first part of the “evidence based practice” equation defined as “best scientific information”. The “practice based evidence” mantra appears to be “it works”, but it begs the more contemporary question “by how much and compared to what?” Of course, positive and negative practice based evidence can coincide with the scientific evidence but this is simply not always the case.
  Finally, chiropractors in the field need to become avid consumers of the evidence provided by good quality research as this will assist lifelong learning and best practice.
9. The profession must support research.
  Research needs to become the number one aspiration of the profession. Research informs both practice and teaching. Without research the profession will not progress. Sadly, the research contribution by the chiropractic profession can only be described as seed like. Figure 1 is a comparison of articles published in the past 45 years by decade using the key words “Physiotherapy” or “Physical Therapy” versus “Chiropractic” (source PUBMED). The Y axis is the number of articles published and the X axis is the decade, the red represents physiotherapy articles, the blue chiropractic. The difference is stark and needs urgent change.

**Fig. 1:**
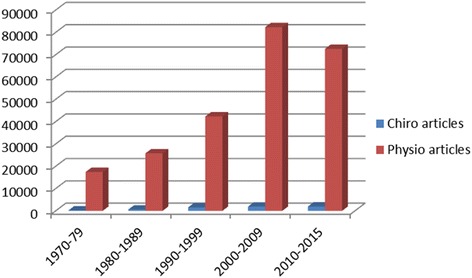
Chiropractic versus Physiotherapy research articles by quantity and decade
  The priorities for chiropractic research need to be examined formally but could revolve around improving clinical practice and patient benefit. This may involve themes such as the accuracy and utility of diagnostic tests, the sub-grouping of people with back and neck pain, prognostic markers, prevention strategies, improving the clinical encounter, the testing of various forms of therapy for safety and effectiveness and importantly dose-response to treatment. This list is not meant to be exhaustive and what is important is that the profession recognises that research is its future life blood and that it be encouraged and resourced immediately and sustainably.
  The training of chiropractors as career researchers needs to be prioritised. All chiropractic staff at Universities and Colleges need to be research active and have or obtain research doctorates such as PhDs. While useful research outputs are evident from many chiropractic colleges it is not enough to have undersized and under resourced research departments within chiropractic programs. It is worth stressing that research is a vital strategy in the advancement of the chiropractic profession and the public it serves.
  If the profession at large ignores research whether in its conduct, administration or its results the profession will wither on the vine. It is acknowledged that the vast majority of practitioners will not undertake research but it is incumbent on all of us to contribute to the research effort with involvement when approached and by regularly contributing funds. If this occurs our involvement and contribution will reward the profession and the public we serve exponentially.
10. Individual chiropractors need to show personal leadership to effect change.
  Change within the profession will likely only occur if individual chiropractors show personal leadership. This necessarily involves providing personal time, developing an individual and/or a collective plan, goal setting for the plan, identifying the obstacles to achieving those goals, taking action to overcome the obstacles, being accountable and providing a concerted long term effort to effect change.
  As part of this personal leadership it will be critical to speak out within the profession. Speak out and become a mentor to less experienced colleagues; speak out and embrace those around you with similar ideals and join them in actively progressing the profession. Do not be diverted for any reason from the worthy goals of the public good, genuinely caring for patients and advancing the profession through your actions and deeds. Your personal leadership is critically important to the profession.

## Conclusion

With the adoption of this ten point plan the chiropractic profession has an opportunity to turn things around within a generation. Importantly, it has an obligation to the public and to successive generations of chiropractors ahead of it. By embracing this plan the profession can be set on a new path, a new beginning and a new direction. This plan should be known as the new chiropractic.
